# Validation of endogenous reference genes for qRT-PCR analysis of human visceral adipose samples

**DOI:** 10.1186/1471-2199-11-39

**Published:** 2010-05-21

**Authors:** Rohini Mehta, Aybike Birerdinc, Noreen Hossain, Arian Afendy, Vikas Chandhoke, Zobair Younossi, Ancha Baranova

**Affiliations:** 1 Molecular and Microbiology Department and Center for the Study of Genomics in Liver Diseases, George Mason University, Fairfax, VA, USA; 2 Translational Research Institute, Inova Health System, Falls Church, VA, USA; 3 Center for Liver Diseases, Inova Fairfax Hospital, Falls Church, VA, USA

## Abstract

**Background:**

Given the epidemic proportions of obesity worldwide and the concurrent prevalence of metabolic syndrome, there is an urgent need for better understanding the underlying mechanisms of metabolic syndrome, in particular, the gene expression differences which may participate in obesity, insulin resistance and the associated series of chronic liver conditions. Real-time PCR (qRT-PCR) is the standard method for studying changes in relative gene expression in different tissues and experimental conditions. However, variations in amount of starting material, enzymatic efficiency and presence of inhibitors can lead to quantification errors. Hence the need for accurate data normalization is vital. Among several known strategies for data normalization, the use of reference genes as an internal control is the most common approach. Recent studies have shown that both obesity and presence of insulin resistance influence an expression of commonly used reference genes in omental fat. In this study we validated candidate reference genes suitable for qRT-PCR profiling experiments using visceral adipose samples from obese and lean individuals.

**Results:**

Cross-validation of expression stability of eight selected reference genes using three popular algorithms, *GeNorm*, *NormFinder *and *BestKeeper *found *ACTB *and *RPII *as most stable reference genes.

**Conclusions:**

We recommend *ACTB *and *RPII *as stable reference genes most suitable for gene expression studies of human visceral adipose tissue. The use of these genes as a reference pair may further enhance the robustness of qRT-PCR in this model system.

## Background

The increasing prevalence of obesity worldwide has drawn research on adipose tissue into the spotlight. Adipose tissue is a complex and highly active tissue with important metabolic and endocrine functions. It not only plays a central role in energy balance but also functions as an endocrine organ secreting various adipokines and cytokines [1,2]. On the basis of its distribution, adipose tissue is divided into three main regions: subcutaneous, intramuscular and visceral fat [3,1].

Accumulation of excessive visceral fat (visceral obesity) is associated with an array of metabolic perturbations including type 2 diabetes, insulin resistance, non-alcoholic fatty liver disease (NAFLD), non-alcoholic steatohepatitis (NASH), cardiovascular disease, hypertension and hyperlipidemia together referred to as "metabolic syndrome" [4,5]. However, the role of visceral obesity in metabolic syndrome is yet to be fully elucidated [6]. Furthermore, a causal relationship between insulin resistance and metabolic syndrome has not been shown conclusively; Obesity seemingly causes insulin resistance, on the other hand insulin resistance appears to aggravate and propagate the adverse effects of obesity [7]. This somewhat co-dependent and circular relationship is difficult to untangle and has generated a multitude of clinical and research publications.

Another area of disagreement involves NAFLD, a common condition affecting about 70% of obese and overweight individuals and increasingly being recognized as a major cause of liver-related morbidity and mortality [8]. The pathological picture of NAFLD encompasses a spectrum of liver injury ranging from simple hepatic steatosis to more severe manifestations, including NASH, which can progress to fibrosis, cirrhosis, and liver failure [9,10]. Studies have reported frequent association of metabolic syndrome and diabetes in patients with NASH, which can progress to NAFLD [9,11,12]. It has even been suggested that hepatic steatosis itself may be the primary cause of insulin resistance and metabolic syndrome in obesity [13]. However, it is still unclear whether NAFLD is a cause or a consequence of insulin resistance [14] and if metabolic syndrome precedes NAFLD or is a result of NAFLD [8]. Many NAFLD centered studies involve the profiling of adipose samples for the production of various soluble mediators of inflammation produced by components of the visceral fat and released in circulation.

Real-time PCR (qRT-PCR) is the standard method for studying changes in relative gene expression in different tissues and experimental conditions. The popularity of this technique is attributed to its high sensitivity and specificity [15]. However, variations in amount of starting material, enzymatic efficiency and presence of inhibitors can lead to quantification errors. Hence the need for accurate data normalization is vital [16]. Among several known strategies for data normalization [17], the use of reference genes as an internal control is the most common approach [15].

An ideal reference gene is one which is consistently expressed at the same level in all samples under investigation regardless of tissue type, disease state, medication or experimental conditions, and exhibits expression levels comparable to that of the target gene [18]. *18S*, β-Actin (*ACTB*), Glyceraldehyde-3-phosphate dehydrogenase (*GAPDH*), Beta-2-microglobulin (*B2M*), RNA polymerase II (*RPII *or *POLR2A*), Tyrosine-3 monooxygenase/Tryptophan-5 monooxygenase activation protein, zeta polypeptide (*YWHAZ*), Ubiquitin C (*UBC*) and Hypoxanthine phosphoribosyl transferase 1 (*HPRT1*) are some of the most commonly used reference genes in RT-PCR studies [2,19,15]. However, numerous studies have shown that expression of these common reference genes vary with tissue type as well as physiological state [20,19]. This variation can potentially explain the often encountered divergence between studies and more seriously, may ultimately result in misinterpretation of data [18]. The suitability of a particular reference gene thus depends on the system being investigated and the inherent experimental conditions [21,19,18].

Recent studies have shown differences in reference gene expression in omental fat tissue between lean and obese patients. In addition there is strong evidence to suggest that obesity and type 2 diabetes mellitus (T2D) exert a detectable influence on reference gene expression in subcutaneous and visceral fat depot [1,2]. In light of these findings, it is crucial for studies involving visceral adipose tissues to validate the stability of the reference genes being used.

Increasing concerns about normalization using ideal reference genes have led to the development of several mathematical algorithms aimed at determining the stability of reference genes [22]. In 2002, Vandesompele et al. have developed the software *GeNorm *that addresses the critical issues of reference gene validation and ranks candidate reference genes according to their expression stability using raw, non-normalized expression levels. Pfaffl et al. have developed similar software, *BestKeeper *that takes into account Ct values of candidate reference genes instead of relative quantities. This software employs a statistical algorithm wherein the Pearson correlation coefficient for each candidate reference gene pair is calculated along with the probability of correlation significance of the pair. Andersen et al. used a model-based evaluation strategy which ranks candidate genes with minimal inter and intra-group variation and developed the software *NormFinder *[23]. In all three softwares, the top ranked genes are recommended for further use in the similar experimental systems as endogenous controls.

In this study we used *GeNorm *[16]. *NormFinder *[23] and *BestKeeper *[24] to validate candidate reference genes suitable for qRT-PCR profiling experiments using visceral adipose samples from obese and lean individuals with and without diabetes.

## Results

To determine the expression stability of eight selected reference genes, RNA expression levels were measured in 19 visceral adipose tissue samples (10 obese visceral adipose tissues and 9 lean visceral adipose tissue samples) and cross-validated using three popular algorithms *GeNorm *v3.4 [16], *NormFinder *[23] and *BestKeeper *[24]. Genes encoding for *18S*, beta-actin (*ACTB*), glyceraldehyde-3-phosphate dehydrogenase (*GAPDH*), beta-2-microglobulin (*B2M*), hypoxanthine guanine phosphoribosyl transferase1 (*HPRT1*), tyrosine 3-monooxygensae/tryptophane 5- monooxygenase activation protein, zeta polypeptide (*YWHAZ*), ubiquitin C (*UBC*) and RNA polymerase II (*RPII*, or *POLR2A*) were selected according to previously published studies that relied on these genes as reference controls [2,19,11]. For each tissue sample, expression stability of each gene was calculated using the mean Ct values. The input data for *BestKeeper *algorithm was raw Ct values, while the analysis using *GeNorm *and *NormFinder *converted raw Ct values to relative quantities using the comparative Ct method [16].

### GeNorm Analysis

**Figure 1:**
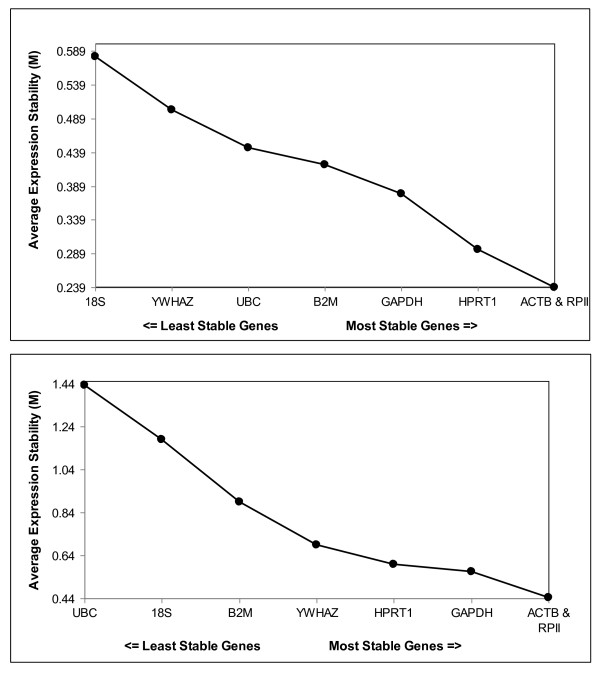
**Gene expression stability M of candidate reference genes in visceral adipose tissue calculated by *GeNorm *software**. The program proceeds with stepwise exclusion of genes with relatively higher variable expression among the samples. The expression stability measure (M) is the average of the stability values of the remaining genes. The lower the M, the more stable the gene in the subset. a) n = 9, b) n = 19.

Investigation of raw non-normalized data of 5 obese and 4 lean visceral adipose tissue samples (n = 9) allowed sorting of genes ranked on the basis of their expression stability (M) from least stable to most stable (*18S *→ *YWHAZ *→ *UBC *→ *B2M *→ *GAPDH *→ *HPRT1 *→ *RPII *and *ACTB*). The respective individual M values compared to the other candidate genes were 0.581, 0.501, 0.446, 0.421, 0.377, 0.295, 0.24 and 0.24 (Figure 1a). Successive elimination of the least stable genes based on the highest M values led to the identification of *ACTB *and *RPII *as the two most stable reference genes. To determine the effect of sample size on the analysis, analysis was done with additional 5 obese and 5 lean visceral adipose tissue samples (n = 19). Yet again *ACTB *and *RPII *were found to be the most stable genes. However the ranking of the least stable and intermediate genes was slightly changed. Sorting of genes from least stable to the most stable revealed UBC as the least stable (*UBC *→ *18S *→ *B2M *→ *YWHAZ *→ *HPRT1 *→ *GAPDH *→ *RPII *and *ACTB*). The respective individual M values compared to the other candidate genes were 1.43, 1.17, 0.88, 0.69, 0.59, 0.56, 0.44 and 0.44 (Figure 1b). Thus increasing sample size did not alter the ranking of the most stable genes indicating the robustness of *GeNorm*.

**Figure 2:**
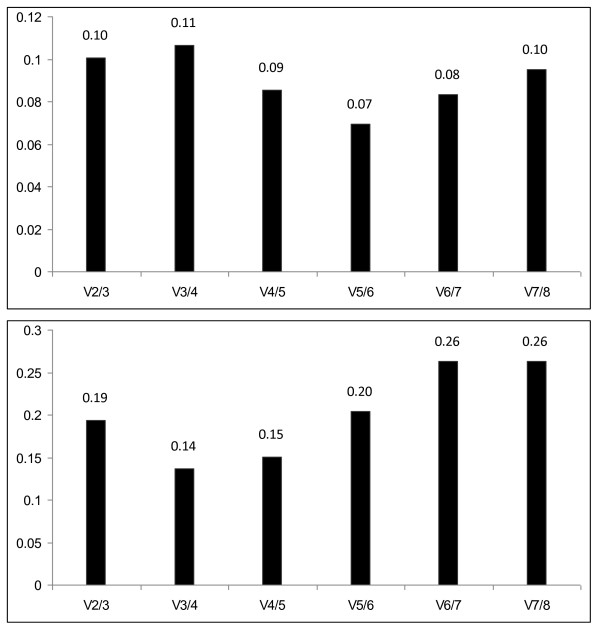
**Determination of optimal number of reference genes for normalization by pairwise variation analysis using *GeNorm *software**. Bar values indicate the magnitude of the change in normalization factor after the inclusion of an additional reference gene. A large variation indicates that the added gene has a significant effect and should probably be included for calculation of the normalization factor. a) n = 9, b) n = 19

Pair-wise variation calculated between two sequential normalization factors (NF_n _and NF_n+1_) for all genes indicates the sufficiency of these two reference genes for accurate normalization (Figure 2a, b).

### NormFinder

**Figure 3:**
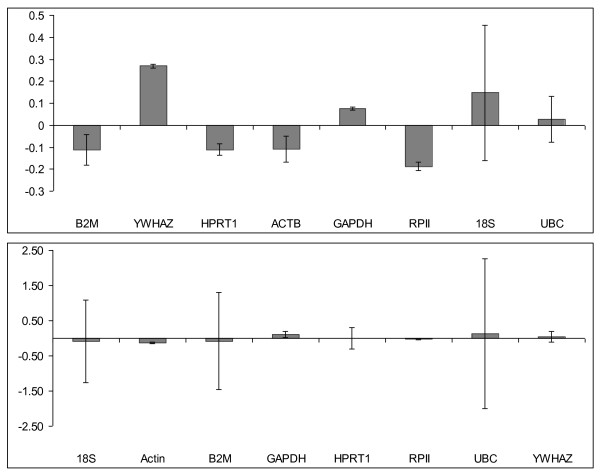
**Determination of the most stable reference genes using *NormFinder***. Two groups considered were - lean and obese patient tissues. Bars represent inter-group variances, while error bars representing the average of intra-group variance. Ideal reference gene has inter-group variation as close to zero as possible and error bars as small as possible. a) n = 9, b) n = 19

Analysis of the gene expression of candidate reference genes in the two subgroups: 5 obese visceral fat and 4 lean visceral fat tissue samples, found *HPRT1 *and *GAPDH *as the two genes with the lowest stability values (Table 1). Further manual inspection for genes with lowest inter group variations showed that although UBC ranked third it had the highest intra-group variation after *18S*. The next two genes in the stability ranking, *B2M *and *ACTB *had similar inter-group variation but *B2M *had slightly higher intra-group variation. *RPII *was ranked below *B2M*, and was found to have a slightly higher inter-group variation and minimal intra-group variation. Thus, using assistance of the process of elimination, *GAPDH*, *HPRT1*, *ACTB*, *B2M *and *RPII *were considered as the best candidate reference genes (Figure 3a). To assess the robustness of this model, sample size was increased to include additional 5 obese and 5 lean visceral adipose tissue samples (n = 19). The ranking, however led to a different set of stable genes. Genes were now ranked similar to *GeNorm *with *RPII *and *ACTB *as the most stable (Table 2). Sorting the remaining genes on basis of inter and intra group variability showed *GAPDH*, *YWHAZ*, *HPRT1 *as the next most stable genes (Figure 3b). Thus *NormFinder *results varied with increase in sample size and with larger sample set the results are in agreement with *GeNorm *analysis.

**Table 1:** Comparison of highly ranked genes by all three software (n = 9).

Gene Name	*BestKeeper Coefficient* *of Correlation (r)*	*GeNorm Expression* *Stability (M)*	*NormFinder Stability* *Value (ρ)*
*ACTB*	0.981	0.239	0.222
*RPII*	0.975	0.239	0.244
*HPRT1*	0.966	0.295	0.193
*GAPDH*	0.915	0.378	0.130
*B2M*		0.421	0.236
*UBC*		0.446	0.207
*YWHAZ*		0.502	0.306
*18S*		0.581	0.344

**Table 2:** Comparison of highly ranked genes by all three software (n = 19).

Gene Name	*BestKeeper Coefficient* *of Correlation (r)*	*GeNorm Expression* *Stability (M)*	*NormFinder Stability* *Value (ρ)*
*ACTB*	0.904	0.45	0.048
*RPII*	0.898	0.45	0.051
*GAPDH*	0.852	0.56	0.097
*YWHAZ*	0.831	0.69	0.109
*HPRT1*		0.60	0.176
*B2M*		0.89	0.382
*18S*		1.18	0.344
*UBC*		1.43	0.468

### BestKeeper Analysis

Unlike *GeNorm *and *NormFinder*, input data for analysis by *BestKeeper *was raw Ct values of each gene. Initial analysis of the data with 9 samples calculated variations (SD (± Ct) and CV (%Ct)) for all the candidate reference genes in the samples, and showed the overall stability in gene expression. None of the candidate reference genes under study showed a SD value higher than 1 indicating that all of the genes under study were suitable to be considered for selection as reference genes.

However, further data processing using pairwise correlation and regression analysis assessed the inter-gene relations and eliminated *18S*, as the gene with the highest variation (CV = 4.36) and least correlation (r = 0.434). The lowest variation was seen for the gene *YWHAZ *(CV = 0.96). However, *YWHAZ *demonstrated only a weak correlation to *BestKeeper *index compared to other candidates (r = 0.653). Therefore, both *18S *and *YWHAZ *were excluded from further analysis. Subsequent elimination singled out *UBC *and *B2M *as genes with low correlation with the *BestKeeper *index. The analysis of the remaining four genes (*HPRT1*, *ACTB*, *GAPDH *and *RPII*) showed a strong and significant correlation (0.914 < r < 0.960) between their expression levels and the *BestKeeper *index (p < 0.001).

To further assess the consistency and reliability of the *BestKeeper *index, sample integrity of all four tightly correlated genes was investigated. The InVar values of all samples were found to have low CP variation as well as low x-fold expression regulation. Ranking the four tightly correlated genes on the basis of variation from the most stable to the least stable was as follows: *GAPDH *→ *HPRT1 *→ *ACTB *→ *RPII*, and yielded the best genes for defining a robust standardizing index. Amongst these four genes *ACTB *and *RPII *were the most highly correlated (0.975 < r < 0.981) to the *BestKeeper *index (Table 3).

**Table 3:** *BestKeeper *correlation analysis (n = 9).

	HPRT1	ACTB	GAPDH	RPII
**Coefficient of Correlation (r)**	0.966	0.981	0.915	0.975
***p *Value**	0.001	0.001	0.001	0.001

In order to determine the influence of sample size on robustness of this algorithm, additional 5 obese and 5 lean visceral adipose tissue samples (n = 19) were included in the analysis. Further data processing using pairwise correlation and regression analysis assessed the inter-gene relations and eliminated *UBC*, as the gene with the highest variation (CV = 10.93) and least correlation (r = 0.350). *18S *gene showed similarly a high variation (CV = 12.99). Therefore, both *UBC *and *18S *were excluded from further analysis. Subsequent sequential elimination based on low coefficient of correlation singled out *B2M *and *HPRT1 *as genes with low correlation with the *BestKeeper *index. The analysis of the remaining four genes (*YWHAZ*, *GAPDH*, *ACTB *and *RPII*) showed a strong and significant correlation (0.831 < r < 0.904) between their expression levels and the *BestKeeper *index (p < 0.001) (Table 4).

**Table 4:** *BestKeeper *correlation analysis (n = 19).

	YWHAZ	ACTB	GAPDH	RPII
**Coefficient of Correlation (r)**	0.831	0.904	0.852	0.898
***p *Value**	0.001	0.001	0.001	0.001

*BestKeeper *calculates the stability measure for each candidate gene and then ranks them from the most to the least stable (SD [± x-fold]). The coefficient of correlation (r) and the p-value measure the correlation between each gene and the *BestKeeper *index. Genes that ranked the best are highlighted.

Ranking the four tightly correlated genes on the basis of variation from the most stable to the least stable was as follows: *YWHAZ *→ *GAPDH *→ *ACTB *→ *RPII*, and yielded the best genes for defining a robust standardizing index. Amongst these four genes *ACTB *and *RPII *were the most highly correlated (0.975 < r < 0.981) to the *BestKeeper *index (Table 2).

## Discussion and Conclusions

To eliminate non-biological variation, gene expression analysis involving qRT-PCR requires stringent normalization strategies. Among the several approaches proposed, use of reference genes is currently the preferred way of normalization [22]. However, the use of improper reference genes is known to lead to erroneous results [25]. Importantly, the studies of the expression levels for the reference gene themselves, particularly, for *GADPH *and *ACTB *[20], showed considerable variation in different tissues and experimental conditions. Specifically in omental and subcutaneous fat depots, a variation in expression of these reference genes was found to be dependent on the presence of obesity and type 2 diabetes mellitus (T2D) [2]. These findings necessitate the need to validate reference genes for studies of human visceral adipose samples.

Scientifically, the validation of reference genes presents a circular problem: assessing stability of expression of a given gene cannot be achieved without using another gene as a reference. Several algorithms have been proposed to address this conundrum [22]. *GeNorm *software [16] is one of the most popular algorithms for validating candidate reference genes with low variability. It utilizes two parameters to quantify the reference gene expression stability: M (average expression stability) and V (pairwise variation). A low M value is indicative of a more stable expression, hence, increasing the suitability of a particular gene as a reference gene. Another feature of *GeNorm *is that it does not require a normal distribution of data. However, co-regulation of candidate genes does seem to influence the efficiency of this algorithm due to the use of pair-wise comparisons. To minimize this risk, the eight candidate reference genes selected for this analysis were chosen on the basis of the difference in their physiological functions-cytoskeleton (*ACTB*), carbohydrate metabolism (*GAPDH*), signaling pathways (*YWHAZ*), transcription (*RPII *or *POLR2A*), metabolic salvaging of nucleotides (*HPRT1*), protein synthesis (*18S*) and protein degradation (*UBC*).

The *GeNorm *algorithm determines expression stability (M) via a pair-wise comparison of one candidate reference gene and all other candidate genes independent of the level of gene expression for each sample. An identical expression ratio of two reference genes in all samples is used as an indicator of expression stability. Thus *GeNorm *analysis is independent of variation in amount of starting material between samples. According to this analysis, *ACTB *and *RPII *represented the best combination of reference genes for visceral adipose tissue among lean and obese patients (Figure 1a), while *HPRT1 *and *GAPDH *were ranked third and fourth, respectively. After completion of this step, a pairwise variation (V) was calculated between two sequential normalization factors (NF_n _and NF_n+1_) for all genes. A large variation indicates that the added gene has a significant effect and should be included for calculation for a reliable normalization factor. Figure 2a, b show that further inclusion of additional reference genes did not significantly increase the pair-wise variation and that the use of two reference genes is sufficient for accurate normalization. An advantage to the *GeNorm *algorithm is that it is minimally affected by expression intensity of the candidate genes. In addition, since the approach is based on multiple pair-wise comparisons, the need for large sample size is mitigated. This was reinforced by the observation that increase in sample size did not dramatically alter the final results. On increasing sample size from 9 to 19, *ACTB *and *RPII *were again found to be the most stable genes with *GAPDH *and *HPRT1 *being ranked the next two best genes (Figure 1b).

Housekeeping genes, in addition to their basic functions, exert pleiotropic effects on other cellular systems, decreasing the value of the function-based predictions of co-regulation. To overcome this problem Anderson et al., proposed a model based approach incorporated into the software *NormFinder*. This algorithm ranks candidate reference genes according to the least estimated intra and inter group variation, which serves as an effective method to overcome the influence of co-regulation. Although *NormFinder *takes into account the heterogeneity in the tested samples, and attempts to distinguish between stability and bias, this model-based approach is self-restricted by the importance it places on overall expression intensity of each candidate gene. A close inspection of the analysis of the results produced by *NormFinder *showed that it biased towards candidate reference genes that have overall similar expression values (in terms of Ct). Consequently, the robustness of this method is linked to the sample size.

The ultimate objective of *NormFinder *is to identify candidate reference genes(s) with an inter group variation as close to zero as possible, while at the same time having small intra-group variation. When the genes were ranked solely by their stability values, *GAPDH *and *HPRT1 *appeared to be the best combination of endogenous controls (Table 1). Further examination of the results reveals that although *UBC *was ranked third, its intra-group variation was large (Figure 3a), therefore, *UBC *gene was eliminated from further consideration leaving the next most stable reference genes: *ACTB*, *B2M *and *RPII*.

The same genes, *GAPDH*, *HPRT1*, *ACTB *and *RPII*, were ranked as the most stable both by *GeNorm *and *NormFinder *softwares. However, the best combinations of two genes proposed by these two algorithms were different. This variation was expected based on the vastly different approaches used by each of the analysis softwares and dependence of robustness of *NormFinder *on sample size. In *GeNorm*, gene expression stability (M) based on the expression ratio of the two genes (pairwise comparison) is the most important criteria for evaluating a reference gene, while *NormFinder *focuses on genes with the least intra and inter group variations. Thus, in cases when two genes show high expression variation while their ratio (M) remains unchanged, there will be discordance in ranking by the two algorithms.

Further, *NormFinder *gains in robustness as the number of samples is increased. This was confirmed by increasing sample size to 19. Ranking of genes from most stable to least stable revealed - *ACTB *and *RPII *as the best combination of genes and this was in accordance with with *GeNorm *results (Figure 3b).

In contrast to the previous study by Catalan et al., in visceral adipose tissue samples both the algorithms highlighted *18S *as one of the least stable gene. This was not unexpected, as several arguments against the use of rRNA as reference genes have been previously put forth. The strongest argument against its use in real time RT-PCR data analysis is its high abundance compared to other target mRNA which hinders accurate subtraction from the baseline value [16].

In order to compare the *GeNorm *and *NormFinder *results with an independent ranking method, the data was also analyzed with the *BestKeeper *tool [24]. In this approach, ideal reference genes are expected to have stable expression, indicated by low variation in the tissue under consideration [24]. With *BestKeeper*, stability (SD) and relationship to the *BestKeeper *index (r and p values) are the two most important criteria for evaluating the stability of reference genes. This algorithm uses a pair-wise correlation analysis for all pairs of candidate genes based on the raw Ct values and calculates the geometric mean of the best suited ones. Based on low CV and high coefficient of correlation (r) to the *BestKeeper *index, *ACTB *and *RPII *followed by *GAPDH *and *HPRT1 *were ranked as the top four genes (Table 3). High correlation coefficient is an indicator of stable expression of the reference genes in visceral adipose tissue. Again, *18S *was ranked as the least stable and excluded from further analysis. Robustness of the algorithm was assessed by increasing sample size (n = 19). Sample size was found to have minimal effect on the results. The same two genes *ACTB *and *RPII *were identified as the most stable followed by *YWHAZ *and *GAPDH *(Table 4).

Overall, the *BestKeeper *results were in line with the *NormFinder *data, and with minor differences, the *GeNorm *data, indicating the reliability of the validation for reference genes in the present study (Table 2). Regardless of the algorithm used, all three software ranked the same set of genes as the most stable.

In conclusion, we recommend *ACTB *and *RPII *as stable reference genes most suitable for gene expression studies of human visceral adipose tissue. The use of these genes as a reference pair may further enhance the robustness of qRT-PCR in this model system.

## Methods

### Samples

Visceral adipose tissue samples were obtained from 10 patients diagnosed with morbid obesity and one of the NAFLD spectrum diseases (n = 10) and nine lean patients with normal liver biopsies (n = 9). Samples were collected at the time of bariatric or other intra abdominal surgery. Samples were flash frozen in liquid nitrogen and stored in -80°C. The samples were de-identified in compliance with HIPAA regulations and this study was approved by Inova IRB.

### Selection of reference genes

Candidate reference genes previously reported as housekeeping genes in adipose tissue were selected as follows: *18S*, beta-actin (*ACTB*), glyceraldehyde-3-phosphate dehydrogenase (*GAPDH*), beta-2-microglobulin (*B2M*), hypoxanthine guanine phosphoribosyl transferase1 (*HPRT1*), tyrosine 3-monooxygensae/tryptophane 5-monooxygenase activation protein, zeta polypeptide (*YWHAZ*), ubiquitin C (*UBC*) and RNA polymerase II (*RPII*, or *POLR2A*).

*ACTB*, *GAPDH*, *B2M *and *HPRT1 *were obtained from Real Time Primers (Real Time Primers, USA). *18S *was synthesized as described previously [26]. Primers for the remaining three genes, *RPII*, *YWHAZ *and *UBC*, were custom designed to span intron-exon boundaries using Oligo Perfect Designer software (Invitrogen, USA) and synthesized commercially (Invitrogen, USA). All primers were confirmed using the NCBI Blast tool against all available mRNA sequences to ensure specificity. Gene accession numbers as well as primer sequences are listed in Table 5.

**Table 5:** Primer sequences of eight reference genes used in the validation study.

Target Gene	Gene Accession Number	Tm	Primer Sequence
B2M	NM_004048.2	55°C	F-5'GTGCTCGCGCTACTACTCTCTCT R-5'TCAATGTCGGATGGATGAAA
GAPDH	NM_002046.2	55°C	F-5'ACAGTCAGCCGCATCTTCTT R-5'GACAAGCTTCCCGTTCTCAG
ACTB	NM_001101.2	55°C	F-5'CTCTTCCAGCCTTCCTTCCT F-5'AGCACTGTGTTGGCGTACAG
HPRT1	NM_000194.1	55°C	F-5'AAGCTTGCTGGTGAAAAGGA R-5'AAGCAGATGGCCACAGAACT
YWHAZ	NM_001135702.1	55°C	F-5'ACTTTTGGTACATTGTGGC R-5'CCGCCAGGAAAAACCAGT
18S	NR_003286.2	60°C	F-5'AGGAATTCCCAGTAAGTGCG R-5'GCCTCACTAAACCATCCAA
RP II	NM_000937.3	60°C	F-5'CTTCACGGTGCTGGGCATT R-5'GTGCGGCTGCTTCCATAA
UBC	XM_002344708.1	60°C	F-5'CCTGGTGCTCCGTCTTAGAG R-5'TTTCCCAGCAAAGATCAACC

### RNA extraction and reverse transcription

Total RNA was extracted from visceral adipose tissues (n = 19) using mirVana RNA extraction kit (Ambion, USA) according to manufacturers protocol. Purity of total RNA was determined as 260 nm/280 nm absorbance ratio with expected values between 1.8 - 2.00 by the GeneQuant1300 spectrophotometer (GE Healthcare, USA). RNA integrity was confirmed by gel electrophoresis using 1% agarose with ethidium bromide [Additional file 1]. 112 ng of extracted total RNA was reverse transcribed using RT^2 ^first strand kit (SABiosciences, USA). According to manufacturer's protocol, total RNA was treated to eliminate genomic DNA and random hexamers and oligo-dT primers were used to prime reverse transcription.

### Quantitative real-time analysis

Quantitative real-time PCR was performed in a 96 well format in the Bio-rad CFX96 Real Time System (BioRad Laboratories, USA). The real-time PCR mixtures consisted of 5 μL cDNA corresponding to ~600 ng total RNA, 0.1 uM of Real-Time primers or 0.2 nM of Invitrogen primer and 1× Sso Fast Evagreen Supermix ( BioRad, USA ) in a final volume of 15 μL. The assay included no template and RT minus controls to detect reagent contamination and presence of genomic DNA. The thermal profile of the RT-PCR procedure repeated for 50 cycles was: 1) 95°C for 10 min; 2)10 s denaturation at 95°C, 40 s annealing at 55°C for Real Time primers and 60°C for Invitrogen primers (amplification data collected at the end of each amplification step); 3) dissociation curve consisting of 10 s incubation at 95°C, 5 s incubation at 65°C, a ramp up to 95°C. (Bio-rad CFX96 Real Time System, USA). Melting curves were used to validate product specificity. All samples were amplified in triplicates from the same total RNA preparation and the mean value was used for further analysis.

### Determination of reference gene expression stability

To assess stability of expression of candidate reference genes across all samples three different statistical algorithms - *GeNorm*, v3.4, *NormFinder *v0.953 and *BestKeeper *v1, were used according to developer's recommendations.

### qRT-PCR GeNorm Analysis

The *GeNorm *tool was used to calculate candidate reference gene stability values (M) using raw non-normalized expression values. For each pair of genes, *GeNorm *calculates a pairwise variation in terms of the standard deviation (V_jk_) of each gene' logarithmically transformed expression ratios (a_ij_) for each tissue sample (m), for any combination of two internal control genes (j or k):

(∀j, k ∈ [1, n] and j ≠ k) [22]:

Expression stability measure (M_j_) is calculated as the mean of pairwise variation of a gene compared to that of all other genes [22].

In iterative steps of exclusion, genes with the lowest expression stability (i.e. the highest M_j _value) are removed. This procedure is repeated until only the genes with the lowest M_j _values and most stable expression remain. The minimum number of genes for which the pair-wise variation V_jk_/V_jk_+1, is smaller than 0.15, is used to define the optimal number of reference genes. The normalization factor is calculated based on the geometric mean of the final optimal set of reference genes.

### qRT-PCR NormFinder Analysis

The *NormFinder *Excel plug-in was used as an alternative algorithm to the *GeNorm *algorithm for determining suitable reference genes in adipose tissue. *GeNorm*, unlike *NormFinder*, uses a model based approach to determine expression stability of control genes. *Normfinder *uses raw non-normalized data in the form of expression values generated using the comparative Ct - method. The *NormFinder *algorithm estimates the overall expression variation of the candidate genes and the variation between sample subgroups using the following model-based approach [23]:

The three components of the model being: the general expression level for candidate gene i within group g (α_ig_), the amount of mRNA in the sample j (β_gj_) and the random variation caused by biological and experimental factors (ε_igj_). The objective is to find the two genes with the least intra- and inter-group expression variation. Confidence intervals on the inter-group variances are indicated by averages of intra-group variances and represented as error bars on inter-group variances. The program algorithm combines the intra-group (σ^2^_ig_) and inter-group (z_ig _- θ_g_, g = 1,...,G) variation and expresses it as a stability value (ρ_ig_) for each investigated gene where γ^2 ^is the variance of expression levels (α_ig_) [23]:

This expression effectively combines multiple sources of variation, and indicates the overall systemic error per gene. Therefore the top ranked gene (which has the smallest stability value, hence the smallest combined variation) is the candidate reference gene most stably expressed in the sample set being investigated. However, since the systemic error value (ρ_ig_) is calculated with the null assumption that expression levels of each gene will be group independent, further manual inspection of inter and intra-group variability was performed. As can be derived from the mathematical expression of the model, this approach gains in robustness as the number of samples is increased.

### qRT-PCR BestKeeper Analysis

*BestKeeper *calculates the gene expression variation for all individual housekeeping genes based on crossing points (CP), as defined by the number of cycles necessary to reach the selected threshold fluorescence [27]. Initial analysis of the data, based on the inspection of raw CP values calculates standard deviation (SD (± CP) and coefficient of variance CV (% CP) for all the reference genes in all of the samples and is used to determine the stability of gene expression. According to the variability observed, reference genes are ranked from the most stable expression: exhibiting lowest variation, to the least stable one with the highest variation. All stably expressed reference genes are combined into an index (*BestKeeper *index) for the respective sample using the geometric mean of each candidate gene's CP values [24].

*BestKeeper *calculates the relationship between each gene by pair-wise correlation analyses, assigning each combination a Pearson correlation coefficient (r) and a probability (p) value. The highly correlated genes are combined into an index and used to calculate the relationship between each candidate reference gene and the index. This serves as an estimate of inter-gene relations and indicates the degree of contribution for each reference gene. Since occurrences of outliers among samples can interfere with the accuracy of the analysis, *BestKeeper *analyses sample integrity (InVar) for differences in respective CP values (n) and for the average CP value of each reference gene (m) [24].

*BestKeeper *then tests each reference gene sample integrity value by subjecting it to the following analysis [24]:

Samples with efficiency corrected intrinsic variation within 3 fold over or under expression are considered acceptable. Hence the *BestKeeper *software seeks to eliminate outliers and thereby increases the reliability and consistency of the *BestKeeper *index.

## Authors' contributions

RM carried out the qRT-PCR studies and drafted the manuscript. ABir designed or extracted primers from previous publications for qRT-PCR and provided daily supervision for RM, a PhD student in training. NH and AA collected visceral adipose samples and extracted relevant clinical information. VC and ZM participated in the design of the study. AB conceived the study, participated in its design, coordinated all efforts and finalized the manuscript. All authors read and approved the final manuscript.

## Supplementary Material

Additional file 1 **An example of the typical result of the gel electrophoresis of total RNA isolated from adipose tissue samples**. An image of the typical agarose gel with total RNA isolated from adipose tissue samples. RNA samples were run on a 1% agarose gel at 100 V for 30 min. Shown here are 6 samples. Samples 1, 2, 3, 4 and 6 show high-quality RNA because there is a clear appearance of the 28S, 18S and 5S rRNA bands. However, sample 5 shows significant degradation and was not used. Click here for file
