# Supplementary material for: Validation of endogenous reference genes for qRT-PCR analysis of human visceral adipose samples
Source: BMC Mol Biol. 2010 May 21;11:39. doi: 10.1186/1471-2199-11-39 (PMC2886049; doi:10.1186/1471-2199-11-39)
Supplement: Additional file 1 — An example of the typical result of the gel electrophoresis of total RNA isolated from adipose tissue samples. An image of the typical agarose gel with total RNA isolated from adipose tissue samples. RNA samples were run on a 1% agarose gel at 100 V for 30 min. Shown here are 6 samples. Samples 1, 2, 3, 4 and 6 show high-quality RNA because there is a clear appearance of the 28S, 18S and 5S rRNA bands. However, sample 5 shows significant degradation and was not used. [file 1471-2199-11-39-S1.DOC]

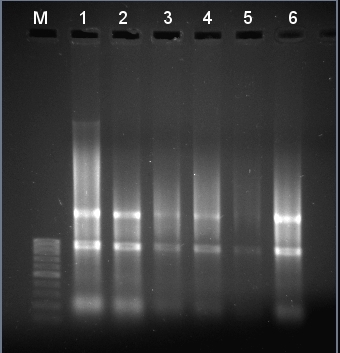


**An example of the typical result of the gel electrophoresis of total RNA isolated from adipose tissue samples.** RNA samples were run on a 1% agarose gel at 100V for 30 min. Shown here are 6 samples. Samples 1, 2, 3, 4 and 6 show high-quality RNA because there is a clear appearance of the 28S, 18S and 5S rRNA bands. However, sample 5 shows significant degradation and was not used.
